# Supplementary material for: Development of novel monoclonal antibodies for detection of pan-Lassa virus
Source: PLoS Negl Trop Dis. 2026 May 11;20(5):e0014326. doi: 10.1371/journal.pntd.0014326 (PMC13175458; doi:10.1371/journal.pntd.0014326)
Supplement: S1 Fig — (DOCX) [file pntd.0014326.s001.docx]

**
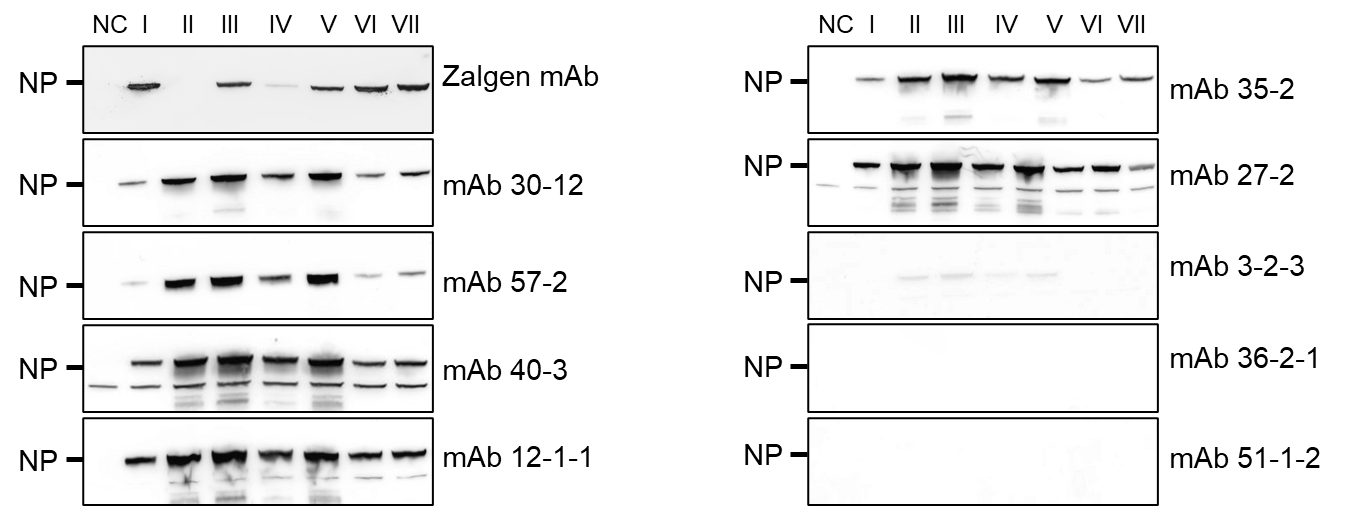
**

**S1 Fig.** Binding of mAbs with cell lysate of HEK293T expressing LASV NPs (lineage I-VII) using WB, lysates were diluted 1:100 times and used as an Ag for measurement of binding of mAbs to LASV NP.
