# Supplementary material for: Development of novel monoclonal antibodies for detection of pan-Lassa virus
Source: PLoS Negl Trop Dis. 2026 May 11;20(5):e0014326. doi: 10.1371/journal.pntd.0014326 (PMC13175458; doi:10.1371/journal.pntd.0014326)
Supplement: S2 Fig — (DOCX) [file pntd.0014326.s002.docx]

**
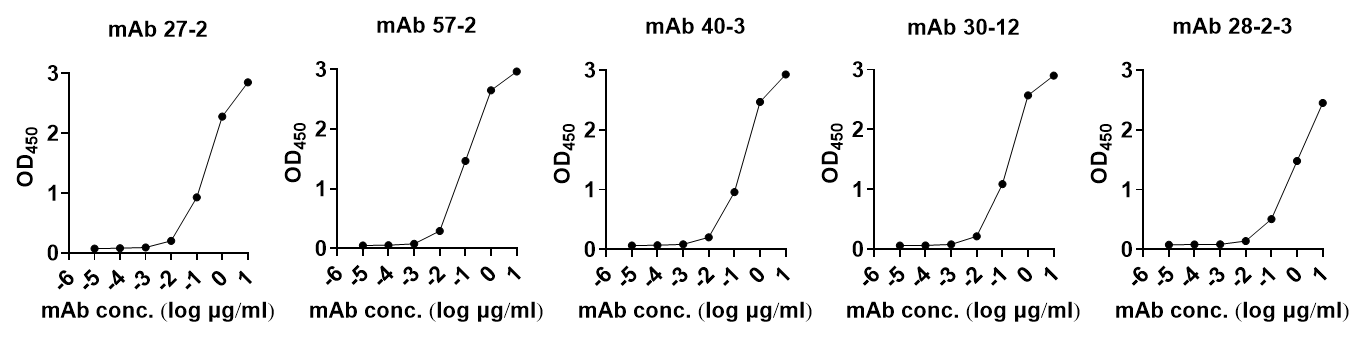
**

**S2 Fig. HRP conjugation of LASV NP mAbs.** The OD values of serially diluted mAbs conjugated with HRP were analyzed by direct ELISA. The lysate of HEK293T expressing LASV Ojoko NP was used as an antigen.
