# Supplementary material for: Development of novel monoclonal antibodies for detection of pan-Lassa virus
Source: PLoS Negl Trop Dis. 2026 May 11;20(5):e0014326. doi: 10.1371/journal.pntd.0014326 (PMC13175458; doi:10.1371/journal.pntd.0014326)
Supplement: S3 Fig — (DOCX) [file pntd.0014326.s003.docx]

**
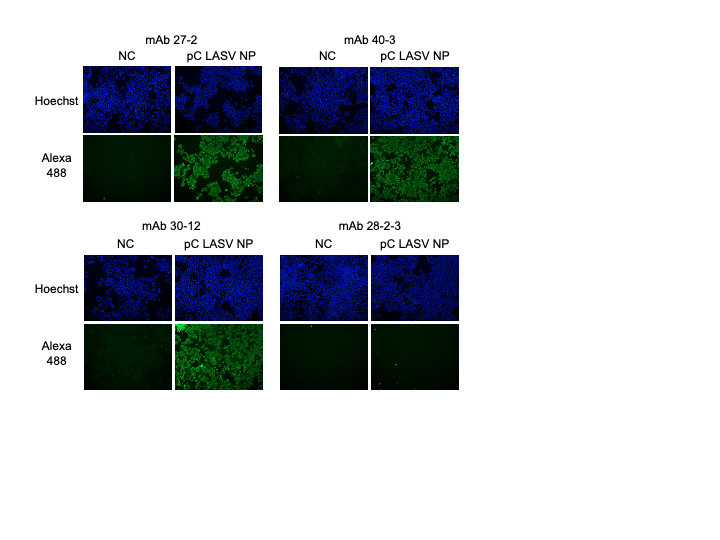
**

**S3 Fig.** IFA showing binding of mAbs to the HEK293T cells expressing LASV NP, 24 hours post transfection cells were fixed, permeabilized and incubated with 27-2, 40-3, 30-12 and 28-2-3 mAb and detected by Alexa488 anti-mouse IgG antibody. Hoechst was used for nucleus staining. HEK293T cells transfected with pCAGGS empty vector were used as negative control (NC).
