# Supplementary material for: Development of novel monoclonal antibodies for detection of pan-Lassa virus
Source: PLoS Negl Trop Dis. 2026 May 11;20(5):e0014326. doi: 10.1371/journal.pntd.0014326 (PMC13175458; doi:10.1371/journal.pntd.0014326)
Supplement: Attachment — Submitted filename: Response to Reviewers_FINAL.docx [file pntd.0014326.s005.docx]

We greatly appreciated the wonderful effort by the reviewers to improve our manuscript. We have revised the manuscript in response to the issues raised by the reviewer, as detailed below. The line numbers listed in the 'Response to Reviewers' section are based on the 'Marked-up Manuscript'.

**Editor Comments:**

**1. Validation in a clinical context: the reviewers emphasized the lack of validation of the developed mAb-based assays using patient samples from endemic areas, as well as the absence of appropriate positive and negative controls. Without these experiments, it is difficult to realistically assess assay performance. As noted, strong results in laboratory-based assays do not necessarily translate to adequate accuracy in clinical settings and more evidence of the clinical relevance of the assay should be provided. Assays should be validated with clinical samples.**

Unfortunately, it is difficult to access the clinical samples during the revision period. However, we added the validation with serum samples from LASV-infected guinea pigs or mice in Table 4. We agree that direct comparison is essential with clinical samples and comparison with other methods. This validation will be performed in the future study. The description of this limitation was added to Discussion section in line 412-418.

**2. Overstated conclusions: In its current form, the manuscript presents conclusions that appear stronger than the data support. These claims should be carefully tempered to remain within the scope of the evidence provided and limitations of the data should be acknowledged in more detail.**

We modified the discussion section according to the reviewers’ suggestions.

**3. Incomplete presentation of mAb data: the reviewers noted that data presented in several Figures are only based on a subset of mAbs, which they found insufficient. A comprehensive presentation of data for all relevant mAbs is required.**

To address this concern, we added the Fig S3.

**Methods:**

**Reviewer #1: Objectives are clearly articulated. Study design is appropriate but incomplete as noted below. Correct statistical analyses used for data presented. No ethical concerns.**

**Response-**

We appreciate the positive feedback from the reviewer regarding our study objectives and study design. We have provided detail response for each comment raised by the reviewer.

**Reviewer #2: Paroha et al. present an interesting study describing the generation and characterization of new monoclonal antibodies against LASV NP from multiple lineages, followed by the development of an antigen-capture sandwich ELISA intended for Lassa fever diagnosis. The methods for antibody generation, screening, and binding characterization (ELISA, Western blotting, and immunofluorescence), as well as identification of non-competing mAb pairs using competitive ELISA, are logical, thorough, and clearly described. The subsequent design of the sandwich ELISA and testing against recombinant antigens, ML29 spiked in serum, LASV isolates from lineages I–IV, and LASV VLPs from lineages V–VII are appropriate for the authors’ stated objectives.**

**That said, the absence of validation using samples from LASV-infected hosts is a major limitation of the methodological framework. While access to acute Lassa fever patient samples is understandably challenging, the authors do appear to have BSL-4 laboratory capacity, and validation using sera from infected animals (e.g., from ongoing or archived challenge studies) would not seem infeasible. Such experiments would provide a more realistic assessment of the assay’s performance in the presence of an active host immune response, clarify whether circulating antibodies might interfere with NP capture, and yield a more accurate estimate of the detection limit. In addition, comparison of assay performance to RT-qPCR would address key questions of sensitivity and specificity. Without these elements, the potential clinical utility of the approach remains uncertain.**

**Response-**

We sincerely thank the reviewer for the detailed and constructive evaluation of our work. The primary objective of the present work was to generate broadly reactive mAbs against LASV NP from different lineages for development of antigen-capture ELISA. For this reason, we focused on comprehensive analytical validation using recombinant antigens, ML29-spiked serum, authentic LASV isolates (lineages I–IV), and VLPs representing other lineages (V–VII). We acknowledge that validation in samples derived from infected hosts is critical for assessing the translational and diagnostic potential of the assay. Therefore, we have evaluated the sandwich ELISA using serum samples obtained from LASV-infected guinea pigs and mice. The assay successfully detected LASV NP antigen in these infected animal sera, demonstrating clear positive reactivity compared to uninfected controls. We have included data in Table 4 and result is updated in result section line 347-351. We agree with the reviewer that host immune responses could influence antigen availability through immune complex formation. However, the positive detection observed in infected animal sera suggests that sufficient free NP could be detected by the mAb pairs.

Regarding comparison with RT-qPCR, we acknowledge that parallel quantitative analysis would further strengthen the diagnostic performance of the assay. While a head-to-head analytical sensitivity and specificity comparison was beyond the scope of the present study, we have clarified this in the revised manuscript line 412-418 that such studies are planned as part of future validation efforts.

**Reviewer #3: Study objectives are clearly stated. The study design does not fully address the stated objective. Statistical details are lacking. See overall comments for details.**

**Response-**

We appreciate the reviewer’s comment and have revised the manuscript to better align the stated objectives with the experimental design, clarifying the scope and intended outcomes of the study. We have provided detailed point-by-point responses below to address these concerns.

**Results:**

**Reviewer #1: Results, including figures, are appropriate except as noted below.**

**Response-**

We have provided detailed point-by-point responses below to address these concerns.

**Reviewer #2: The analyses presented are consistent with the described methods and are explained clearly and concisely. The results are well structured, and the figures and tables are of good quality, with clear labeling and appropriately detailed legends that make the data easy to interpret. Overall, the presentation of the results is strong. I have two minor suggestions for improvement:**

1) **In Figure 3C (lines 304–307), if multiple antibody pairs were tested against ML29-spiked serum, it may be clearer to display the results from all pairs, as in Figure 3B, rather than only showing the 40-3 + 57-2 pair. This would allow the reader to better evaluate relative assay performance.**
2) **In the Discussion (lines 401–405), the authors mention western blot results assessing cross-reactivity of the mAbs against other arenaviruses, but these data are not shown. While this experiment is peripheral to the main objectives, including the blots in the Supplementary Information (if permissible) would provide completeness and transparency.**

**Response-**

**1)** We have tested animal LASV infected serum samples with sandwich ELISA and our data suggested that mAbs generated in this study can be used to detect LASV in serum samples. Therefore, we have removed the data with ML29 spiked serum samples in Fig 3C and corresponding result.

**2)** We have deleted the sentences related to the cross-reactivity against other arenaviruses because that is out of scope this study. Instead, we added LCMV data (Fig. 4C) and modified the paragraph in lines 354 and 366.

**Reviewer #3: The analysis matches the objectives, but is incomplete. Comprehensive presentation of data is lacking. The figures and tables are of sufficient quality.**

**Response-**

We thank the reviewer for the positive assessment of the figure and table quality. We acknowledge the concern that the data presentation was not sufficiently comprehensive. In response, we have revised the manuscript to include additional experimental details, expanded datasets where applicable and incorporated quantitative analyses to ensure a more complete and transparent presentation of the results.

**Conclusions:**

**Reviewer #1: Conclusions are appropriate. Limitations are well noted. Public health relevance is addressed.**

**Response-**

We appreciate this assessment and are pleased that these aspects of the manuscript are clear and appropriately presented.

**Reviewer #2: The development of improved diagnostics for Lassa fever remains an important and unmet public health need, particularly for tools that can be deployed in resource-limited community clinics and at the point of care. This study contributes meaningfully by identifying novel broadly reactive monoclonal antibodies and demonstrating their application in an antigen-capture ELISA format. The findings represent a potentially valuable step toward diagnostic tools with utility across diverse LASV lineages.**

**At the same time, the conclusions somewhat overstate the likely impact of the assay as presented. Other groups have previously developed antigen-capture ELISAs and rapid diagnostic tests (RDTs) for LASV that performed well in controlled laboratory studies but ultimately showed inadequate sensitivity and specificity in field evaluations—limitations that explain why RT-qPCR remains the diagnostic gold standard in endemic regions. The authors briefly cite these mixed outcomes, but they do not provide a clear rationale for why their assay is likely to perform differently or better in real-world settings. For example, most clinical evaluations of Zalgen’s ReLASV Pan-Lassa RDT were conducted in Nigeria and Sierra Leone, where lineages II, III, and IV predominate. Thus, the poor field performance of that test was unlikely due to restricted lineage coverage. More broadly, antigen-based assays face inherent constraints, such as reduced sensitivity later in infection when circulating antibodies begin to complex with free antigen. This context underscores that broader cross-lineage detection, while valuable, may not itself overcome the central challenges of sensitivity and specificity in clinical practice (see, for example, Chi et al., PLoS Glob Public Health, 2025; Elsinga et al., Lancet Infect Dis, 2024; Boisen et al., Sci Rep, 2020; Boisen et al., Sci Rep, 2018).**

**To strengthen their conclusions, I suggest that the authors explicitly acknowledge these limitations and outline the steps needed to establish the assay’s utility. In particular, side-by-side comparisons of their mAbs against existing antigen-capture platforms, coupled with validation against RT-qPCR (the diagnostic gold standard), will be essential. Such experiments, ideally performed using clinical or animal challenge samples, would provide critical evidence that this assay represents a meaningful improvement over current tools.**

**Response-**

As the reviewer mentioned that previous antigen-detection platforms-including the ReLASV *Pan*-Lassa RDT-demonstrated promising analytical performance but did not consistently achieve a promising clinical applicability. In response, we have revised the discussion section in line 412-418, we agree that direct comparative studies will be essential.

**Reviewer #3: The conclusions are not fully supported by the data as presented. The limitations are only moderately described.**

**Response-**

We have revised the manuscript to include additional experimental details, expanded datasets where applicable and revised the result and discussion of this study. Limitations of the study have been updated in discussion section in lines 412-418.

**Editorial and Data Presentation Modifications:**

**Reviewer #1: Minimal line editing needed.**

**Response-**

We have carefully proofread the revised manuscript to ensure clarity, consistency and accuracy.

**Reviewer #2: The manuscript is generally well organized, and the figures and tables are clear and appropriately annotated. However, there are several places across the manuscript where grammar, syntax, and sentence structure issues make the text difficult to follow. I suggest that the authors carefully review the full manuscript to address these issues and improve clarity and readability, which would strengthen the overall presentation of their findings.**

**Response-**

We have revised the manuscript and carefully proofread the revised manuscript to ensure clarity, consistency and accuracy.

**Summary and General Comments:**

**Reviewer #1: In their manuscript entitled, “Development of novel monoclonal antibodies for detection of pan-Lassa virus”, Paroha and colleagues describe the generation of anti-LASV NP mAbs with cross reactivity for all seven LASV lineages and the development of an antigen-capture sandwich ELISA for pan-LASV detection. An unknown number of mice were immunized with virus like particles. Approximately 2000 hybridomas were created and screened. Of these, 61 screened candidates were tested for reactogenicity to LASV NP from all seven lineages. Of these, 10 proved cross-reactive. Competition assays were performed to identify mAbs with non-overlapping epitopes. An antigen-capture sandwich ELISA was then developed using optimized pairs of non-completing mAbs. Overall, this is a very well written manuscript. The methods and results are very clearly written and understandable.**

**As noted below, I have some minor comments that should be addressed. My greatest concern is the lack of positive and negative controls mentioned in the text. While the ELISA development was clearly described, it is not useful without positive controls. Furthermore, the authors have yet to describe the next critical step in the assays development which is testing it using human samples from endemic areas from LASV suspected patients with both positive and negative results by another methodology (e.g. pcr). Even data using a panel of retrospective samples would be adequate to give an initial determination of sensitivity and specificity. As written, the mAb and assay development are incomplete.**

**Other Comments:**

**1. Methods: Overall, the description of the methods is quite complete and well written. However, in the description of the ELISAs and immunofluorescence assays there is no mention of positive or negative controls. What was used? Please describe these as well as the number of replicates used in each experiment and the reproducibility of the results.**

**2. Why were 4 of the mAbs not tested by IFA?**

**3. Was a non-LASV mAb used as a negative control to ensure no non-specific binding was occurring?**

**4. Line 306: What serum was used? Human or mouse?**

**5. Lines 341-343: I understand why rVSV, EBOV and MARV were used to identify non-specific binding. It would also be useful to know if there is binding to LCMV.**

**6. Line 383: The word sensitivity should not be used here as no studies to determine sensitivity were presented. LOD would be more appropriate.**

**7. Lines 396 -408: The potential cross-reactivity with other arenaviruses should be moved to the results section and the data presented. LCMV should be included as well.**

**Response-**

We agree that appropriate positive and negative controls are essential for ELISA validation. We have used positive control antibody from Zalgene, which reportedly shown to react with LASV NP, however our WB data in supplementary figure S1 showed that this positive control mAbs cannot detect all the seven lineages, therefore this positive control antibody was not considered a control for our experiments. To date, no single mAb has been reported that recognizes the LASV NP across all seven known LASV lineages. Because our study specifically aimed to develop broadly reactive mAbs capable of detecting NP from all seven lineages, no previously available universal antibody could serve as a positive control for cross-lineage detection using which we can validate our ELISA system with. Instead, we used negative controls which included cell lysate from HEK293T expressing empty vector and we have further verified our sandwich ELISA by testing non-LASV samples.

Regarding validation with serum samples, we have evaluated the sandwich ELISA using serum samples obtained from LASV-infected guinea pigs and mice. The assay successfully detected LASV NP antigen in these infected animal sera, demonstrating clear positive reactivity compared to uninfected controls. We have included data in Table 4 and result is updated in result section line 347-351.

Our responses to other comments are as follows-

**1)** As mentioned above no universal antibody could serve as a positive control for cross-lineage detection using which we can validate our ELISA system. Therefore, we did not use positive control in our experiment.

2) We have now included the IFA results for the additional mAbs in supplementary information in S3 Fig. These data demonstrate that the tested mAbs bind to LASV NP in IFA-based analyses except for 28-2-3 mAb. Although 28-2-3 mAb produced a clear band at the expected molecular weight in western blot analysis, no detectable signal was observed in IFA. This discrepancy is likely due to differences between denaturing (WB) and native/fixed (IFA) conditions. The antibody may recognize a linear epitope exposed under denaturing conditions but inaccessible in the fixed protein. Furthermore 28-2-3 mAb showed non-competitiveness with other mAbs, which also strengthened the idea that this mAb is binding with other epitopes on LASV NP. Additionally, fixation-induced epitope masking or limited antigen accessibility could also be the lack of detection in IFA with 28-2-3 mAb.

**3)** We thank the reviewer for this important question. In the present study, we did not include a non-LASV mAb as a negative control. Instead, depending on assay, ELISA specificity was assessed using either appropriate antigen-negative controls, including cell lysate from HEK293T expressing empty vector or virus control such as VSV, or non-LASV such as EBOV and MARV and LCMV which consistently showed no detectable signal. These controls allowed us to confirm that the observed reactivity was LASV NP antigen-dependent rather than due to non-specific binding of the detection system.

4) The serum used in this experiment was human serum. Since we have revised our result with LASV serum data therefore we have removed ML29 spiked serum data from this study. Accordingly, we have deleted Fig 3C and corresponding result in line 309-312.

5) We thank the reviewer for this insightful suggestion. We agree that assessing potential cross-reactivity with LCMV is important given its taxonomic relationship to LASV. In response to this comment, we have now evaluated the binding of our mAbs against LCMV and updated the results section accordingly in Lines 354 and 366. The data demonstrates that no cross-reactivity was observed. These results have also been included in Fig 4C. These findings further support the specificity of the developed mAbs for LASV NP detection.

6) We have updated the information in Line 397.

7) As described above, we deleted these sentences and modified the paragraph in line 438. As per reviewer’s suggestions, we have also updated figure 4C with LCMV result in lines 354 and 366.

**Reviewer #2: This study reports the generation of broadly reactive monoclonal antibodies against LASV nucleoprotein and their application in a sandwich ELISA. The work is carefully executed, the experiments are appropriate to the stated objectives, and the data are presented clearly. These reagents could make a useful contribution to ongoing efforts to develop improved diagnostics for Lassa fever, which remains a pressing public health need.**

**The principal limitation is the absence of validation with serum or blood samples from LASV-positive hosts. While the binding results with purified virus isolates and VLPs are convincing, there is ample published evidence showing that strong performance in controlled laboratory assays does not necessarily translate into adequate sensitivity and specificity in clinical or field settings. Without testing in animal or patient samples—or a much clearer rationale for why this assay is likely to perform better than previous tools—the broader significance of the findings is difficult to assess.**

**In its current form, the study offers valuable preliminary data and novel reagents. However, to substantiate the conclusions, additional testing (discussed above) is strongly encouraged. If such experiments are not feasible at this stage, the conclusions should at minimum be framed more cautiously, with greater emphasis on the next steps needed to establish clinical relevance and real-world utility.**

**Response-**

We thank the reviewer for the thoughtful and constructive comment. We agree that evaluation of the assay using serum or blood samples from LASV-positive hosts is essential to establish clinical sensitivity and specificity. As noted, our current study primarily focused on the generation and analytical characterization of broadly reactive mAbs against LASV NP and the development of sandwich ELISAs using recombinant proteins, VLPs, and purified virus isolates. Furthermore, to support our findings, we have performed ELISA using serum samples form LASV-infected guinea pigs and mice. The assay successfully detected LASV NP antigen in these infected animal sera, demonstrating clear positive reactivity compared to uninfected controls. We have included data in Table 4 and result has been updated in line 347-351.

**Reviewer #3: Paroha, et al, address the need for improved diagnostic methods for Lassa Fever (LF), which are currently complicated by the genetic diversity of the virus. The authors generated a panel of monoclonal antibodies (mAbs) targeting the LASV nucleoprotein (NP). Several mAbs showed broad reactivity across LASV lineages I-VII in ELISA and western blot and immunofluorescence assays. Antigen-capture assays were then used to identified pairs of mAbs that could be used in diagnostics. While the premise is reasonable, the lack of comprehensive data presentation and controls, and unjustified conclusions about the sensitivity and clinical applicability is a major weakness. Without addressing these concerns, the impact of the study and the potential of the developed ELISA for improved LF diagnosis remain uncertain.**

**Key concerns include:**

**1) There is incomplete data presentation in Figs 1, S1, S2 and Fig 3. Data is shown for only a subset of the mAbs. The authors should include comprehensive data presentation for all mAbs, or at the minimum, those that are bolded in Table 1. Only one mAb pair is presented in Fig 3. It is unclear whether the other mAbs pairs were also tested in this format or if they did not perform well. Regardless, all data should be presented.**

**2) Critical details are missing regarding ELISA data analysis, including the quantification method for lysates to ensure equal coating of plates, the basis for LOD determination and controls used to set this LOD. Controls in general are lacking throughout the experiments.**

**3) Reactivity levels are misrepresented in Table 1. For example, mAb 57-2's western blot reactivity to LI, LVI, and LVII is weaker than to LII-V, but all are indicated as "+," giving a false impression of uniform reactivity.**

**4) The signal for the mAb pair 30-12/28-2-3 is at the LOD line. The data presented don't support determination of 6.2E2 as the sensitivity for this pair.**

**5) The lineage-specific data presented in Figure 4 is focused on virus diluted in serum-free DMEM when viral-spiked sera controls would have been more impactful for measuring clinical relevance. The signal for the ML29-reassortment is reduced from a maximum of 1.2 in the absence of sera to 0.5 in the presence of sera. The maximum signals for live LASV were 0.4-0.9 in the absence of sera. Presumably, the same reduction in signal would occur in the presence of sera, calling into question the true sensitivity for live Lassa.**

**6) Line 305-307: The authors claim of “reliable detection of virus in sera” based on a single replicate with one mAb pair is an overstatement based on the limited analyses and replication of experiments.**

**7) The authors state that “… the detection methods using these novel mAbs can detect unknown LASV belonging to another lineage or LASV-like arenaviruses that cause viral hemorrhagic fever”. The data supporting this claim is not presented. If the authors mean that sequence conservation suggests the mAbs might detect LASV-like arenaviruses, then the statement needs clarification.**

**Response-**

1) We have updated the IFA result shown in Fig 1C, in the revised manuscript we have included the results of IFA data from 27-2, 40-3, 30-12 and 28-2-3 mAbs in S3 Fig. Result is updated in line 257. All the mAbs have shown positive binding with LASV NP except 28-2-3 mAb, we believe due to different epitope binding 28-2-3 shown positive binding in WB but did not show IFA signal, we have responded this comment above. S1 and S2 Figs already contained all the mAbs used in the sandwich ELISA system and were highlighted in bold in Table 1. Fig 3C have been removed, as we have updated the result with serum testing using sandwich ELISA in Table 4.

2) In the current study, total protein concentrations of lysates were not directly measured prior to plate coating. Instead, we used standardized volumes of lysates prepared under identical conditions and ELISA plates were coated with equal volume of lysate in each well. To ensure assay reliability and reproducibility, appropriate negative control was included on every plate in all the ELISA experiments. LOD for each assay was calculated based on the results (positive or negative) using a threshold value, mean plus three standard deviations of the negative control. As described above, while no universal *pan*-lineage positive control antibody exists, use of appropriate negative controls in different experiments ensured that the observed ELISA signals were antigen-specific and reproducible.

3) We agree that the western blot band intensities for mAb 57-2 is different between different lineages. However, the purpose of Table 1 was to indicate qualitative reactivity of tested mAbs (i.e., presence or absence of detectable binding) rather than to provide a quantitative comparison of different mAbs for each LASV lineages. mAbs showing WB signal above background were denoted as positive (“+”), while those did not react to LASV NP were denoted as (“-”). Importantly, different viral lineages may express varying levels of the NP, which can also influence western blot band intensity independent of antibody binding affinity. Thus, variations in signal strength do not necessarily reflect differences in intrinsic antibody reactivity. As the primary objective of this study was to generate mAbs suitable for development of an ELISA system capable of detecting the antigen across different lineages of LASV, quantitative comparison of western blot signal intensity was not the focus of this work.

4) We would like to clarify the difference between threshold of ELISA detection and LOD. The dotted line represents the threshold of the sandwich ELISA, which is the cut-off used to define positive versus negative signal. While LOD was calculated based on the X-axis concentration of antigen that produced a signal distinguishable from background according to standard LOD calculation methods. These are two different measures: the dotted line indicates the assay cut-off, while the LOD reflects the minimal detectable virus titre. For the 30-12/28-2-3 mAb pair, although the signal at low antigen concentrations approaches the threshold, the LOD was determined using the defined calculation method, which yielded the reported value of 6.2×10² PFU.

5) To address this, we have now performed the sandwich ELISA using animal serum samples. In these assays, all tested mAb pairs for LASV isolates, produced detectable positive signals in the presence of serum, confirming that the assay functions in a biologically matrix. As expected, the absolute signal was somewhat reduced compared to serum-free conditions due to low virus titre in infected animal serum samples. However, the assay reliably detected LASV NP at concentrations consistent with our previously calculated LOD. We have updated the results section in Table 4 and line 347-351 to include these serum-based data, providing a more realistic assessment of assay sensitivity and clinical relevance.

6) We have removed Fig 3C and corresponding result and updated serum results in Table 4. As demonstrated by our serum data from animal samples that mAbs pair with 28-2-3 and 57-2 could detect LASV in serum samples effectively in both guinea pig and mice serum samples, while other pairs of mAbs did not detect the lower virus titre in serum samples. Therefore, we have also revised the discussion section that among tested mAbs pairs 28-2-3 and 57-2 mAbs pair has shown better reactivity when tested with serum samples in line 409-411.

7) To address the reviewer’s concern, we rephrased the sentence in line 431-435.
