# Supplementary material for: Development of novel monoclonal antibodies for detection of pan-Lassa virus
Source: PLoS Negl Trop Dis. 2026 May 11;20(5):e0014326. doi: 10.1371/journal.pntd.0014326 (PMC13175458; doi:10.1371/journal.pntd.0014326)
Supplement: Attachment — Submitted filename: Response to Reviewers_2nd revise FINAL.docx [file pntd.0014326.s006.docx]

We appreciated the reviewers comments. Accordingly we have revised the manuscript in response to the issues raised by the reviewer, as detailed below. The line numbers listed in the 'Response to Reviewers' section are based on the 'Marked-up Manuscript'.

**Reviewer #2: While the serum samples from infected guinea pigs and mice are appropriately referenced to prior studies, it would be helpful to include a brief summary of key experimental details (e.g., virus strain, infection route, timing of collection) either in the Methods, Results, or table legend. This would allow readers to better interpret the findings without needing to consult the original references.**

**Response-**

We agree that providing these details improves the clarity and accessibility of the study. As suggested, we have added a concise summary of the experimental parameters for the animal models including the specific viral strains used, routes of infection, and sample collection timelines in the method section lines 225-227 and line 371.

**Reviewer #2: The use of infected animal sera is a valuable addition; however, these models may not fully recapitulate antigen dynamics observed in human infection. It would be helpful to ensure that these data are clearly framed as supportive of analytical performance rather than direct evidence of clinical diagnostic utility (lines 355-358).**

**Response-**

We thank the reviewer for this insightful comment. We agree that while the infected animal models demonstrate the assay's ability to detect native antigen in a complex biological matrix, they do not perfectly mirror the kinetics of human infection.

As suggested, we have revised the text in result section line 342-343 and 346-349 to clarify that these data serve as proof of analytical performance and robustness in a physiological context, rather than a direct claim of clinical diagnostic utility. We have also further added a statement regarding the need for future validation with human clinical samples.

**Reviewer #2: The discussion of potential cross-reactivity with other arenaviruses is scientifically relevant; however, given the limited geographic overlap between LASV and New World arenaviruses, the practical diagnostic implications of this point may be more limited than implied (lines 433-438).**

**Response-**

We have revised the discussion (Lines 423-425).

**Reviewer #2: There are still minor issues with grammar and sentence structure throughout the manuscript. A final round of careful language editing would improve clarity and readability**

**Response-**

We acknowledge the reviewer’s concern regarding the clarity and readability of the manuscript. We have conducted a thorough, line-by-line review of the entire text to correct grammatical errors and improve sentence structure. Specific attention was paid to the Results and Discussion sections to ensure that technical descriptions are precise and easy to follow. These correction can be easily tracked in track on manuscript file.
